# Positive Theory of the Fecundation of the Mammiferæ

**Published:** 1845-03

**Authors:** 


### BIBLIOGRAPHICAL NOTICES.

In the American Journal of the Medical Sciences for January, we find a review of several recent works upon the new views respecting fecundation and menstruation. The review embodies so much that is interesting and important, that we take the liberty of presenting our readers with a partial abstract, regretting that our limits will not permit us to make more copious extracts. The works reviewed are as follows:

"*Positive Theory of the Fecundation of the Mammiferæ.* By F. A. Pouchet, M. D., Paris, 1842."

"*Of Puberty and the Critical Age in Women, and of the Periodical Discharge of Ova by Women and the Mammiferæ.* By M. A. Raciborski, M. D. &c., Paris, 1844, pp. 520."

